# Knowledge mining of brain connectivity in massive literature based on transfer learning

**DOI:** 10.1093/bioinformatics/btae648

**Published:** 2024-12-05

**Authors:** Xiaokang Chai, Sile An, Simeng Chen, Wenwei Li, Zhao Feng, Xiangning Li, Hui Gong, Qingming Luo, Anan Li

**Affiliations:** Britton Chance Center for Biomedical Photonics, Wuhan National Laboratory for Optoelectronics, Huazhong University of Science and Technology, Wuhan 430074, China; Britton Chance Center for Biomedical Photonics, Wuhan National Laboratory for Optoelectronics, Huazhong University of Science and Technology, Wuhan 430074, China; Britton Chance Center for Biomedical Photonics, Wuhan National Laboratory for Optoelectronics, Huazhong University of Science and Technology, Wuhan 430074, China; Britton Chance Center for Biomedical Photonics, Wuhan National Laboratory for Optoelectronics, Huazhong University of Science and Technology, Wuhan 430074, China; Key Laboratory of Biomedical Engineering of Hainan Province, School of Biomedical Engineering, Hainan University, Haikou 570228, China; Key Laboratory of Biomedical Engineering of Hainan Province, School of Biomedical Engineering, Hainan University, Haikou 570228, China; HUST-Suzhou Institute for Brainsmatics, JITRI, Suzhou 215123, China; Britton Chance Center for Biomedical Photonics, Wuhan National Laboratory for Optoelectronics, Huazhong University of Science and Technology, Wuhan 430074, China; HUST-Suzhou Institute for Brainsmatics, JITRI, Suzhou 215123, China; Key Laboratory of Biomedical Engineering of Hainan Province, School of Biomedical Engineering, Hainan University, Haikou 570228, China; Britton Chance Center for Biomedical Photonics, Wuhan National Laboratory for Optoelectronics, Huazhong University of Science and Technology, Wuhan 430074, China; Key Laboratory of Biomedical Engineering of Hainan Province, School of Biomedical Engineering, Hainan University, Haikou 570228, China; HUST-Suzhou Institute for Brainsmatics, JITRI, Suzhou 215123, China

## Abstract

**Motivation:**

Neuroscientists have long endeavored to map brain connectivity, yet the intricate nature of brain networks often leads them to concentrate on specific regions, hindering efforts to unveil a comprehensive connectivity map. Recent advancements in imaging and text mining techniques have enabled the accumulation of a vast body of literature containing valuable insights into brain connectivity, facilitating the extraction of whole-brain connectivity relations from this corpus. However, the diverse representations of brain region names and connectivity relations pose a challenge for conventional machine learning methods and dictionary-based approaches in identifying all instances accurately.

**Results:**

We propose BioSEPBERT, a **bio**medical pre-trained model based on **s**tart-**e**nd position **p**ointers and **BERT**. In addition, our model integrates specialized identifiers with enhanced self-attention capabilities for preceding and succeeding brain regions, thereby improving the performance of named entity recognition and relation extraction in neuroscience. Our approach achieves optimal F1 scores of 85.0%, 86.6%, and 86.5% for named entity recognition, connectivity relation extraction, and directional relation extraction, respectively, surpassing state-of-the-art models by 2.6%, 1.1%, and 1.1%. Furthermore, we leverage BioSEPBERT to extract 22.6 million standardized brain regions and 165 072 directional relations from a corpus comprising 1.3 million abstracts and 193 100 full-text articles. The results demonstrate that our model facilitates researchers to rapidly acquire knowledge regarding neural circuits across various brain regions, thereby enhancing comprehension of brain connectivity in specific regions.

**Availability and implementation:**

Data and source code are available at: http://atlas.brainsmatics.org/res/BioSEPBERT and https://github.com/Brainsmatics/BioSEPBERT.

## 1 Introduction

Neuroscientists have long been engaged in the endeavor to map connectivity across the entire brain. Recent developments in virus tracers, optical imaging, and computing techniques have significantly expanded the scope of research in this field (Oh *et al.* 2014, Zhou *et al.* 2023). The Allen Mouse Brain Connectivity Atlas (AMBCA) (Oh *et al.* 2014), for instance, furnishes a brain-wide connectivity map, facilitating the computation of interconnectivity relations among 213 brain regions based on data from 469 experiments, thus constructing a cortico-striatal-thalamic network. Although existing research has accumulated a great deal of knowledge about brain connectivity, our understanding of whole-brain connectivity remains fragmented, with much of the research concentrated on specific brain regions. The neuroscience literature holds a vast repository of information regarding brain region connections, and leveraging literature mining techniques can aid in mapping brain connectivity. In recent years, researchers have manually charted brain connectivity in select brain regions. For instance, Bota *et al.* (2015) compiled data from over 16 000 rat cortical reports utilizing a modularity algorithm to cluster 73 cortex regions, thereby identifying 4 distinct classes of modules. Similarly, Swanson *et al.* (2017) analyzed 32 350 connection reports expertly collated from published experiments, revealing a network of macroconnections among 77 cortical regions. They obtained a stable bihemispheric six-module solution through network community detection, albeit at the cost of approximately 4000 h for data collection and organization. Relying on manual mapping of brain region connectivity proves to be not only time-consuming and labor-intensive but also necessitates ongoing maintenance efforts.

With recent advances in deep learning and biomedical text mining, the automatic mining of information from biomedical literature has become a significant area of research. Soto *et al.* (2019) developed Thalia, a semantic search engine that recognizes eight different types of concepts in biomedical abstracts. Similarly, Wei *et al.* (2019) introduced PubTator Central, a text-mining system designed to retrieve genes, proteins, diseases, and other biomedical entities from articles. However, there is still a gap in existing research, as no comprehensive text mining system currently addresses brain region entities and connectivity relations.

Extracting neuroanatomical regions (also known as brain regions) from the literature is a pivotal task in constructing brain connectivity maps. However, there are numerous brain regions in the literature with varying vocabularies, leading to several challenges in automatic extraction. These challenges include the use of acronyms, synonyms, and different nomenclatures for the same word. For example, the Anterior Hypothalamic Nucleus is denoted as AHN in Allen's nomenclature, AHA in Hof's nomenclature, and AH in Paxinos' nomenclature. The CA1 field of the hippocampus is a term commonly used in literature. It should be noted that CA1 and hippocampus may be identified as separate entities. French *et al.* (2009) annotated 18 242 brain regions from 1377 articles in the Journal of Comparative Neurology (JCN), creating the first large gold-standard corpus of brain regions. They used linear chain conditional random fields (CRF) with model features based on morphological, lexical, syntactic, and contextual information to achieve an F1 score of 78.6% on the gold-standard corpus. Richardet *et al.* (2015) proposed the BraiNER relying on linear chain CRF and additional species information, to achieve an F1 score of 81.6%. Shardlow *et al.* (2019) achieved a state-of-the-art F1 score of 81.8% for brain region recognition using the Bi-LSTM-CRF and bi-directional semantic features of text sequences. Although some progress has been made in recognizing brain regions, the evaluation results are lower than the lowest 83.1% F1 score achieved for other types of entities in PubTator Central (Wei *et al.* 2019), an automated annotation system for the biomedical domain.

In neuroscience, it is essential to accurately recognize the connectivity and directional relations between brain regions. This task can help neuroscientists organize fragmented knowledge about connectivity. French *et al.* (2012) created the WhiteText connectivity corpus, which contains 989 articles from the JCN, with 4338 sentences. They achieved a 58.4% F1 score on the corpus by using the All-paths graph kernel method. Richardet *et al.* (2015) proposed the BraiNER method to achieve a 64% F1 score on the corpus. They extracted 226 993 connectivity relations between brain regions from abstracts and full-text articles. Sharma *et al.* (2021) used the BioBERT model (Lee *et al.* 2020) and achieved a state-of-the-art F1 score of 75%. In addition, understanding the inputs and outputs of neuronal circuits requires clear directionality of connectivity relations. Gökdeniz *et al.* (2016) extracted PVT connectivity from 14 manually annotated PVT cases using predefined patterns and syntactic analysis to determine directional relations. They achieved an F1 score of 58.39% and extracted 811 relations from 558 publications (451 abstracts and 107 full-text articles) containing 343 different brain regions. Although previous work has made progress in automatically extracting connectivity and directional relations from the literature, there are still a significant number of misclassifications in these areas.

In this study, we propose BioSEPBERT, a pre-trained model based on start-end position pointers. The proposed BioSEPBERT model outperforms existing state-of-the-art models in brain region recognition, connectivity relation extraction, and directional relation extraction tasks. We create a corpus for directional relations using a defined rule and back-to-back manual annotation for the paraventricular nucleus of the thalamus (PVT) directional relations. This corpus serves as a valuable resource for training and evaluating models in directional relation extraction tasks. This improvement demonstrates the efficacy of BioSEPBERT in accurately capturing and extracting knowledge from neuroscience literature. Utilizing the BioSEPBERT model, we extract 22.6 million standardized brain regions and 165 072 directional relations from abstracts and full-text articles. This data is used to construct a comprehensive knowledge graph of brain region connectivity relations, enabling the validation and analysis of input-output circuit knowledge for any given brain region. Moreover, it facilitates efficient retrieval of connectivity knowledge from the entire literature corpus concerning input-output circuits of specific brain regions.

## 2 Materials and methods

### 2.1 Datasets

We assess different models using two benchmark corpora focusing on brain region names and connectivity relations: the WhiteText corpus and the WhiteText connectivity corpus (French *et al.* 2012). The WhiteText corpus comprises annotations from 1377 JCN articles, encompassing 17 585 brain region annotations, with a 90.7% annotation agreement rate between two annotation experts. Before brain region recognition, the WhiteText corpus is converted to the CoNLL format (Sang and De Meulder 2003). The brain region words are tagged as ‘B-BrainRegion’ for the first word and ‘I-BrainRegion’ for subsequent brain region words, while other words are labeled as ‘O’. The corpus is divided into training, validation, and test sets, maintaining a ratio of 6:1:1, facilitating 8-fold cross-validation assessments. In the WhiteText connectivity corpus, connectivity relations are annotated within 4338 sentences extracted from 989 abstracts mentioning two or more brain regions. These relations are classified into two categories: those with connectivity relations (totaling 3097) and those without (totaling 19 475). The WhiteText connectivity corpus is divided into training, validation, and test sets at an 8:1:1 ratio. We partitioned the data into document-level and sentence-level corpora to investigate the impact of data distribution on models.

We establish a corpus of brain region directional relations (Table 1) sourced from the WhiteText connectivity corpus (French *et al.* 2015). The corpus undergoes manual annotation by two domain experts and encompasses a total of 6511 sentences extracted from 2817 abstracts. Among these sentences, there are 5201 connected relations, comprising 2441 output connections, 947 input connections, and 1813 undirected connections, alongside 29 196 unconnected relations. Remarkably, the two labeling experts reach a consensus on 92.3% of the connectivity relations' labeling.

**Table 1.** Three different types of corpora used in the experiment.

Dataset task	Size	Types	Counts
Named entity recognition	1377 abstracts	Brain region	17 585
Relation extraction	989 abstracts	Connectivity	22 572
	2817 abstracts	Directivity	34 397

### 2.2 Position pointer-based named entity recognition model for brain regions

Brain region NER constitutes a sequence annotation task. Previous approaches have either entailed manual feature augmentation, as seen in the Linear Chain CRF (French *et al.* 2009), or automatic feature extraction from static word vectors like Word2Vec (Tomas *et al.* 2013), using deep learning architectures such as Bi-LSTM-CRF (Shardlow *et al.* 2019). Recent efforts have shifted toward utilizing semantic features extracted from vast biomedical literature using pre-trained models, which are then fine-tuned for specific downstream tasks. Examples include BioBERT (Lee *et al.* 2020), PubMedBERT (Gu *et al.* 2021), SciBERT (Beltagy *et al.* 2019), and ClinicalBERT (Alsentzer *et al.* 2019), among others (Luo *et al.* 2022). Building upon this foundation, we present three distinct methodologies for brain region extraction.

The first method, MTM-CW (Wang *et al.* 2019), is based on Bi-LSTM-CRF. We engage in co-training brain region names alongside gene, protein, disease, and chemical names, leveraging multi-model parameter sharing to improve brain region recognition. The second method entails using pre-trained models within the biomedical domain, namely BioBERT v1.1 (cased, 12 layers, corpora from PubMed, vocabulary size 28996), PubMedBERT (uncased, 12 layers, abstracts and full-text from PubMed and PMC, vocabulary size 30522), SciBERT (uncased, 12 layers, papers from Semantic Scholar, vocabulary size 31090), and ClinicalBERT (cased, 12 layers, notes from MIMIC, vocabulary size 28996). Leveraging pre-trained parameters gleaned from biomedical literature, we fine-tune these models on the brain region corpus to facilitate brain region prediction. The optimal hyperparameter settings for the pre-trained models are detailed in Supplementary Table S1. Lastly, we propose BioSEPBERT for brain region recognition. BioSEPBERT leverages name boundary features via position pointers and acquires word semantic features through pre-trained models.

**Figure 1.**
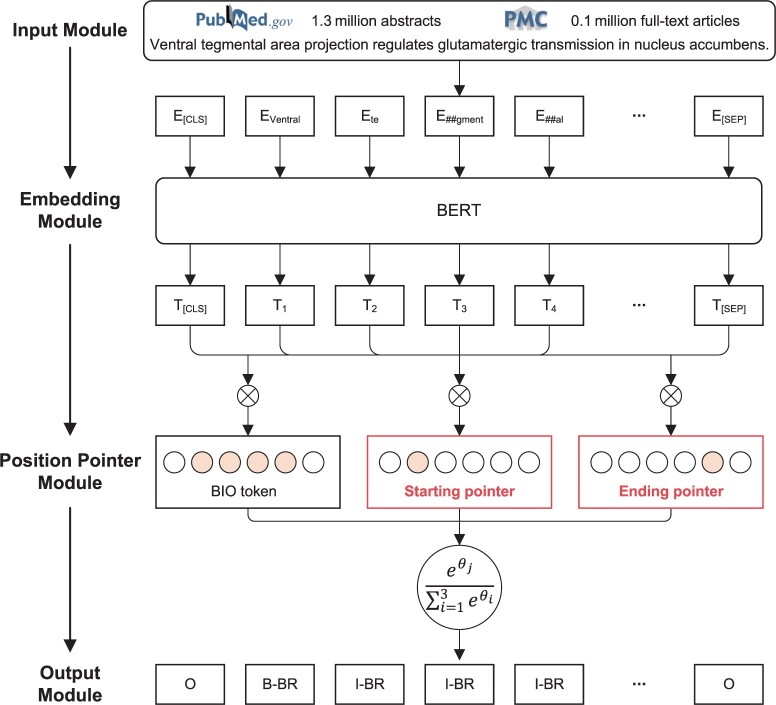
The structure of BioSEPBERT for brain region NER. The model incorporates start and end position pointers subsequent to the embedding module. These pointers are strategically used to enhance the model's capability in learning features related to brain region boundaries.

The structure of the BioSEPBERT is depicted in Fig. 1, segmented into distinct modules: the input module, embedding module, position pointer module, and output module. In the input module, $S=<s_{1},s_{2},\ldots ,s_{n}>$, where $s_{i}\;\left(1\leq i\leq n\right)$ represents the words from the text of PubMed abstracts (PubMed) and PubMed Central full-text articles (PMC), and $Y=<y_{1},y_{2},\ldots ,y_{n}>$, where $y_{i}\;\left(1\leq i\leq n\right)$ represents the categorization labels assigned to the words. To ensure accurate word recognition, each word is tagged with start and end pointers, denoted as *T* and *D*. These tags are encapsulated within $T=<t_{1},t_{2},\ldots ,t_{n}>$ and $D=<d_{1},d_{2},\ldots ,d_{n}>$. After encoding in the input module, the sentence *S* is represented as a token sequence $X=<x_{1},x_{2},\ldots ,x_{n}>$, where each token $x_{i}$ (for 1≤i≤n) is represented as

(1) $$x_{i}=x_{i}^{w}+x_{i}^{p}+x_{i}^{s}$$

where $x_{i}^{w}$ is the pre-trained word embedding, $x_{i}^{p}$ is the position embedding, $x_{i}^{s}$ is the segmentation embedding, and $x_{i}\in R^{H}$.

In the embedding module, all parameters are taken from the PubMedBERT model, and the structure of transformer is taken from Vaswani *et al.* (2017), and the formula is computed as follows:

(2) $$O^{0}=W_{p}X+b_{p}$$

(3) $$O^{l}=\mathrm{Trans}\left(O^{l-1}\right)$$

where X is the embedding of the tokens, $W_{p}$ and $b_{p}$ are the parameters of the PubMedBERT model. $\mathrm{Trans}\left(x\right)$ represents the transformer block and x represents the input vector. $l\;\left(1\leq l\leq L\right)$ denotes the current layer. L denotes the number of the transformer layers. After the embedding module, the output is represented as $O=<o_{1},o_{2},\ldots ,o_{n}>$, where $o_{i}\in R^{H}$.

In the position pointer module, the training result is decoded into three components: $P^{l}$, $P^{s}$, and $P^{e}$, representing the label prediction, the start position prediction, and the end position prediction, respectively. The formula is computed as follows:

(4) $$P^{i}=\mathrm{sigmod}(W_{i}O_{i}+b_{i})$$

where $P^{i}$ represents the label prediction $P^{l}$, the start position prediction $P^{s}$, and the end position prediction $P^{e}$.

In the output module, the loss function of the training process aims to minimize three types of cross-entropy functions:

(5) $$L=-\frac{1}{N}\left[\begin{array}{c} \sum_{i=1}^{N}\sum_{j=1}^{M_{1}}y_{ij}\;\text{log}\;\left(p_{ij}^{l}\right)+\\ \lambda \left(\sum_{i=1}^{N}\sum_{j=1}^{M_{2}}s_{ij}\;\text{log}\;\left(p_{ij}^{s}\right)+\sum_{i=1}^{N}\sum_{j=1}^{M_{2}}e_{ij}\;\text{log}\;\left(p_{ij}^{e}\right)\right) \end{array}\right]$$

*N* represents the total number of samples, $M_{1}$ and $M_{2}$ represent the number of label types and position-pointer types. The variables $y_{ij}$, $s_{ij}$, and $e_{ij}$ are 0 or 1, indicating whether class label *j* is the correct classification for sample *i*, respectively. Similarly, the variables $p_{ij}^{l}$, $p_{ij}^{s}$, and $p_{ij}^{e}$ represent the probability of the model predicting class label *j* for sample *i*, respectively. λ represents the coefficient of the position-pointer.

### 2.3 Position pointer-based relation extraction model for brain region relations

Extracting relations between brain regions is essentially a categorization task. Except for the input and position pointer modules, other modules bear a resemblance to those utilized in NER models. We present three distinct models tailored for extracting connectivity relations: recurrent neural networks [e.g. Att-BLSTM (Zhou *et al.* 2016)], pre-training models (such as BioBERT, PubMedBERT, etc.), and BioSEPBERT.

**Figure 2.**
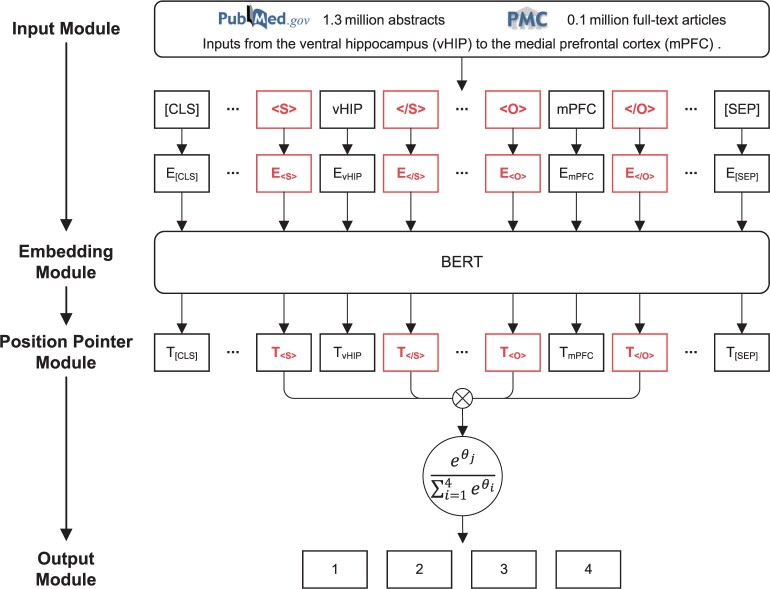
The structure of BioSEPBERT for brain region RE. The model strategically extracts various entity features and relation semantic features in terms of start and end position pointers to strengthen the influence of entity name, entity position, and relation features on classification.

The BioSEPBERT relation extraction model, as illustrated in Fig. 2, incorporates boundary pointer labels (<s>, </s>) and (<o>, </o>) for the front and back brain regions within the input module. The input words are denoted as follows:

(6) $$X=\left\{c_{1},x_{1},\ldots ,l_{11},x_{e1},l_{12},\ldots l_{21},x_{e2},l_{22},\ldots x_{n},c_{2}\right\}$$

where $c_{1},\;c_{2}$ represent the special identifier [CLS] and [SEP], $x_{e1}$, $x_{e2}$ denote the different entities, and $l_{11},\;l_{12},\;l_{21},\;l_{22}$ represent different boundary pointer labels.

In the embedding module, BERT represents PubMedBERT model for encoding the token sequence X.

After the embedding module, the output is denoted as $O=<o_{1},o_{2},\ldots ,o_{n}>$, where $o_{i}\in R^{H}$. From the output vectors, we obtain the boundary pointer vectors $s_{1},s_{2}{,e}_{1},e_{2}$. These boundary pointer vectors are merged with the name features of the front and back brain regions, the location features, and the sentence semantic features to calculate the classification output probability of each sentence:

(7) $$P\left(X\right)=W_{o}C\mathrm{oncat}\left(\begin{array}{c} W_{1}^{s}s_{1}+b_{1}^{s},\;W_{2}^{s}s_{2}+b_{2}^{s},\\ W_{1}^{e}e_{1}+b_{1}^{e},W_{2}^{e}e_{2}+b_{2}^{e} \end{array}\right)+b_{o}$$

where $W_{1}^{s},W_{2}^{s},W_{1}^{e},W_{2}^{e}\in R^{M\times H}$ represent the weight of $s_{1},s_{2}{,e}_{1},e_{2}$. $b_{1}^{s},b_{2}^{s},b_{1}^{e},b_{2}^{e}\in R^{M}$ represent the bias of $s_{1},s_{2}{,e}_{1},e_{2}$*. M* represents the number of class types. $W_{o}\in R^{M\times 4M}$, $b_{o}\in R^{M}$ are the weights of the output vector.

In the output module, the training process is to minimize the multi-classification cross-entropy function:

(8) $$L=-\frac{1}{N}\left(\sum_{i=1}^{N}\sum_{j=1}^{M}y_{ij}\;\text{log}\;\left(p_{ij}\right)\right)$$

*N* represents the total number of samples. The variable $y_{ij}$ is 0 or 1, indicating whether class label *j* is the correct classification for sample *i*, and the variable $p_{ij}$ represents the probability of the model predicting class label *j* for sample *i*.

### 2.4 Brain region connectivity knowledge graph

Constructing a brain region connectivity knowledge graph involves three key steps: acquiring literature articles, extracting entities and relations, and mapping entities to an ontology.

First, literature articles are acquired using the Entrez Programming Utilities (E-utilities) (Wheeler *et al.* 2008) API to obtain text from PubMed and PMC. Common terms from brain region ontologies, including Allen (Sunkin *et al.* 2013), NeuroNames (Bowden and Dubach 2003), and the Brain Architecture Management System (BAMS) (Bota and Swanson 2008), along with their abbreviations and full names, are provided to the E-utilities API to retrieve the PMIDs of all related literature, which are then downloaded and stored. This process ensures that the knowledge base includes all relevant brain region literature, as E-utilities also search for synonyms of the provided terms.

Next, multiple types of entities and relations are extracted from the literature. The literature text is tokenized according to Equation (1) after clause and word splitting, then input into the BioSEPBERT model to identify brain region entities and their locations within the text. To extract brain region relations, we select all statements containing two or more brain region entities, convert them to the form shown in Equation (6), and input them into the BioSEPBERT relation model to determine whether an association exists between any pair of brain region entities. Other types of entities, such as species, genes, and diseases, can be extracted by inputting PMIDs into PubTator Central (Wei *et al.* 2019).

Finally, the extracted brain region entities are mapped to an ontology. Due to limitations in ontology coverage, exact matching alone often fails to map all brain region entities accurately. To address this, we develop a fuzzy matching method that enhances exact matching by incorporating single-word matching and weighted sorting based on word-matching scores. For entities that cannot be matched precisely, we either remove directional words or use ScispaCy (Neumann *et al.* 2019) for nested entity recognition before attempting to match. If the entity still cannot be matched, it is split into individual words, each of which is compared one by one with ontology terms, including their synonyms. The match rate between the entity and the ontology terms, along with their synonyms, is calculated, and a score is assigned based on the weights. This approach allows us to select the best matching ontology term, significantly improving the mapping rate for brain region entities.

## 3 Results

### 3.1 Brain region extraction

**Figure 3.**
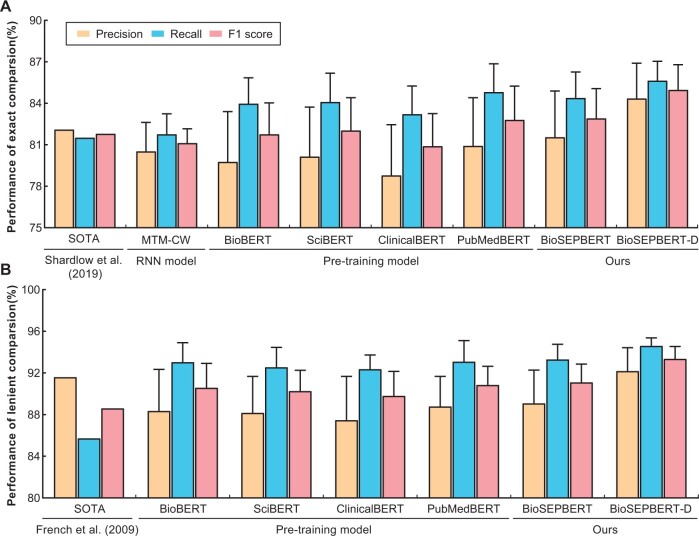
Two types of evaluation of different models in brain region recognition. The height of the error bar denotes the standard deviation. (A) shows the performance of different models under exact matching. (B) shows the performance of different models under lenient matching.

The WhiteText corpus serves as the evaluation benchmark for three categories of models. Evaluation is conducted through 8-fold cross-validation, using two distinct metrics: exact matching, wherein predicted brain regions must precisely match annotated ones, and lenient matching, allowing predicted boundaries to be smaller or equal to annotated regions. The results of the evaluation for the three model types are shown in Fig. 3.

The state-of-the-art models achieve F1 scores of 81.8% (Shardlow *et al.* 2019) and 88.6% (French *et al.* 2009) for exact and lenient matching, respectively. The first model, recurrent neural networks MTM-CW, exhibits lower performance compared to the state-of-the-art model under exact matching. Despite parameter sharing among multiple entities such as genes, diseases, and chemicals, MTM-CW fails to significantly enhance brain region recognition. The second class of models are pre-trained models in the biomedical domain like BioBERT, SciBERT, and PubMedBERT. Trained on abstracts or full-text articles conducive to brain region recognition, PubMedBERT emerges as the frontrunner, achieving an optimal F1 score of 82.8% and 90.9% under both exact and lenient matching, respectively. This represents a 1.2% and 2.5% improvement over the state-of-the-art model, respectively. In contrast, ClinicalBERT, pre-trained on the clinical dataset MIMIC-III (Johnson *et al.* 2016), proves less suitable for brain region recognition, yielding a lower F1 score of 80.9% under exact matching when compared with other pre-trained models. The third model, the pre-trained position pointer-based model BioSEPBERT, surpasses PubMedBERT by integrating boundary pointer features. By incorporating dictionary tree-based denoising features, BioSEPBERT achieves an optimal F1 score for brain region recognition, reaching 85.0% and 93.4% for exact and lenient matching, respectively. This marks an improvement of approximately 2.6% and 2.8% over PubMedBERT, respectively. Detailed data can be found in Supplementary Table S2.

We conduct further analysis to compare the effects of the position-pointer, corpus size, and corpus quality on BioSEPBERT (Table 2). Data augmentation is achieved through corpus size expansion via repetition of the training set, while data denoising is facilitated by using the dictionary tree method. Notably, the dictionary tree-based denoising feature emerges as the most influential factor in the model’s performance. It improves the exact F1 score from 82.9% to 84.7% and the lenient F1 score from 91.1% to 93.0%. This underscores the pivotal role of corpus quality in enhancing brain region recognition. In addition, the position-pointer features ensure that the model’s lower bound remains unaffected. However, the sole addition of data augmentation fails to improve the model’s performance and reduces its effectiveness. However, when combined with other features, such as data denoising, it contributes to an improvement in the exact F1 score.

**Table 2.** Evaluation of different features of BioSEPBERT in the brain region recognition.^a^

Group	Feature			Performance	
	Position-pointer	Augmentation	Denoising	Exact F1 (%)	Lenient F1 (%)
1	√			82.90 ± 2.17	91.10 ± 1.76
2		√		82.15 ± 2.47	90.32 ± 2.07
3			√	84.74 ± 2.41	93.00 ± 1.08
4	√	√		83.03 ± 0.96	90.20 ± 1.46
5	√		√	84.81 ± 2.16	**93.35 **±** **1.13
6	√	√	√	**84.97 **±** **1.83	**93.35 **±** **1.20

a The data is represented as mean ± SD, with the best scores highlighted in bold.

### 3.2 Brain region connectivity and directional relations extraction

**Figure 4.**
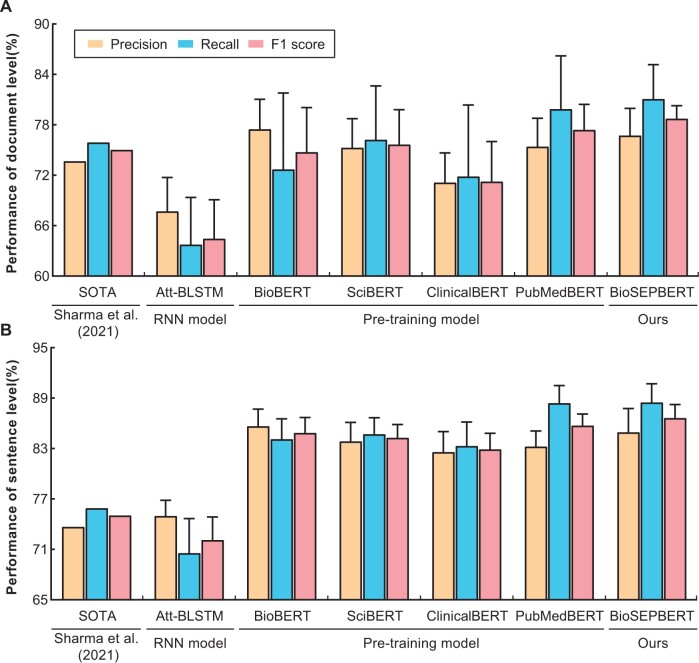
Evaluation of different models in connectivity relation extraction. The height of the error bar denotes the standard deviation. (A) shows the performance of the models at the document level. (B) shows the performance of the models at the sentence level.

Three types of models are used to evaluate the WhiteText connectivity corpus, which is divided into document-level and sentence-level corpora. Various models, including Att-BLSTM, BioBERT, and PubMedBERT, among others, have been replicated for evaluation. The results of these evaluations on both corpora are shown in Fig. 4.

The state-of-the-art model achieves an F1 score of 75% in connectivity relation extraction (Sharma *et al.* 2021). All models demonstrate superior performance at the sentence level compared to the document level, indicating a significant influence of varying data distributions on model effectiveness. Optimal results are achieved when training and test sets exhibit congruent sample distributions. Several pre-trained models within the biomedical domain achieve F1 scores ranging between 82%–86%, surpassing the current state-of-the-art model. These models exhibit precision in extracting connectivity relations between brain regions. Our BioSEPBERT model achieves F1 scores of 78.7% and 86.6% for the two corpora, surpassing the best results of PubMedBERT by 1.7% and 1.1%, respectively. The incorporation of special identifiers for start and end pointers furnishes additional positional and semantic information regarding brain region connectivity relations. BioSEPBERT outperforms other models at the document level, a feat that exceeds the performance of other models at the sentence level. Detailed data can be found in Supplementary Table S3.

Previous studies have primarily concentrated on identifying the presence of connectivity between brain regions in literature, neglecting to account for the directionality of such connectivity. In our study, we redefine connectivity relations into four distinct types: no connection, connections lacking directionality, input connections, and output connections. Within the directional relation corpus, we observe 2441 output connections, 947 input connections, 1813 undirected connections, and 29 196 unconnected relations. Please refer to the Materials and Methods section for the details.

A 10-fold cross-validation methodology is used to evaluate the various models (Table 3). The results reveal that Att-BLSTM only attains a modest F1 score of 61.4% in directional relation extraction, suggesting that recurrent neural networks may not be well-suited for extracting domain-specific directional relations. Conversely, several pre-trained models consistently achieve F1 scores of 83%–86%, mirroring the performance observed in connectivity relation extraction. BioSEPBERT outperforms PubMedBERT in directional relation extraction, achieving an F1 score of 86.5%, representing a 1.1% improvement. We compare the confusion matrices of BioSEPBERT and PubMedBERT for directional relation extraction. BioSEPBERT achieves normalized metrics of 98%, 80%, 92%, and 88% for unconnected, undirected, output, and input relations, respectively. In comparison, PubMedBERT achieves normalized metrics of 98%, 84%, 86%, and 87% for the same categories. Notably, BioSEPBERT shows a 7.0% improvement in output connections and a 1.1% improvement in input connections, suggesting that our method is more effective in extracting input-output connectivity relations from the literature. Detailed data are provided in Supplementary Table S4.

**Table 3.** Performance of different models in the directional relation extraction.^a^

Model	Precision (%)	Recall (%)	F1 score (%)
Att-BLSTM	66.98 ± 4.24	58.21 ± 3.93	61.42 ± 2.29
BioBERT	84.09 ± 1.69	85.07 ± 1.46	84.51 ± 1.17
SciBERT	85.00 ± 2.09	85.30 ± 2.53	85.09 ± 1.95
ClinicalBERT	82.67 ± 1.54	84.15 ± 1.71	83.38 ± 1.40
PubMedBERT	84.73 ± 1.99	86.58 ± 1.07	85.60 ± 1.34
BioSEPBERT	**86.33 **±** **1.74	**86.73 **±** **1.30	**86.50 **±** **1.46

a The data is represented as mean ± SD, with the best scores highlighted in bold.

Our BioSEPBERT model consistently achieves optimal results in both connectivity relation extraction and directional relation extraction tasks. The results indicate that BioSEPBERT is capable of extracting knowledge about connections between brain regions from large-scale literature.

### 3.3 Construction of a knowledge graph in brain region connectivity

The proposed approach lends itself to the construction of a comprehensive brain region connectivity knowledge graph by extracting brain region entities, connectivity relations, and directional relations from a corpus comprising a vast number of abstracts and full-text articles. All brain region entities are normalized to a standard dictionary, ensuring preservation of all matches.

The extraction of connectivity knowledge from both abstracts and full-text articles is integral to the knowledge graph construction process (Table 4). Initially, PubMed and PMC are queried using commonly used brain region names, yielding a substantial dataset comprising 1.3 million abstracts and 193 100 full-text articles. Subsequently, the BioSEPBERT model is used to extract 6.7 million brain region entities from 1.3 million abstracts, with an average of 5.0 brain region entities per abstract, 28 406 connectivity relations, 29 513 output connectivity relations, and 8845 input connectivity relations. Meanwhile, our model extracts 23.7 million brain region entities from 193 100 full-text articles, with an average of 123.2 brain region entities per full-text article, 85 997 connectivity relations, 93 568 output connectivity relations, and 33 955 input connectivity relations. The number of brain region entities identified in a single full-text article surpasses that of a single abstract by >24 times, underscoring the wealth of information contained within full-text literature. PubTator Central extracts 3.4 million species entities from abstracts and 11.9 million species entities from full-text articles. Among the 3.4 million abstracts, the most frequently mentioned species were humans, rats, and mice, with occurrences of 1.5 million, 0.8 million, and 0.4 million, respectively. Analyzing species distribution assists in categorizing connectivity relations according to species.

**Table 4.** Statistical information on brain regions, connectivity, and directional relations in the literature.

Knowledge graph statistics	PubMed abstracts	PMC full-text articles
Documents	1 349 979	193 100
Brain regions	6 780 131	23 792 888
Entity link brain regions	5 042 023	17 585 149
Connectivity relations	28 406	85 997
Input relations	8845	33 955
Output relations	29 513	93 568
Species	3 416 381	11 991 754

In constructing the brain region connectivity knowledge graph, brain region entity normalization serves as a crucial step. Existing brain region ontologies such as Allen, NeuroNames, and BAMS each encompass distinct anatomical names, abbreviations, and synonyms for different species. To consolidate these varied ontologies, we selected data for mice and rats from the three widely used ontologies: Allen, NeuroNames, and BAMS. By performing exact matches on full names and abbreviations, supplemented by manual correction, yielding a comprehensive dictionary of brain regions comprising 2354 unique IDs. During the normalization process, entities extracted from the literature are linked to this dictionary utilizing both exact and fuzzy matching techniques. Within the 1.3 million abstracts, only 3.4 million brain region entities successfully match the entries in the dictionary, leaving approximately 3.3 million entities unlinked. To address this issue, we develop a fuzzy matching method predicated on single-word matching and weighted sorting based on word-matching scores. This method facilitates the linking of entities, resulting in an additional 1.6 million brain region entities being matched, thereby enhancing the efficacy of literature-mining endeavors. Details of the ontology mapping evaluation process can be found in Supplementary Table S5.

The construction of the brain region connectivity knowledge graph involves the extraction and linking of brain region entities, alongside the extraction of connectivity and directional relations between them. Compared to previous research on mining knowledge about brain regions, our methodology not only achieves superior accuracy in extracting entities and relations but also leverages a more comprehensive normalized ontology dictionary and refined entity-linking method. This study marks the first instance of extracting connectivity relations between brain regions with both input and output directions from literature. Our method holds the potential to chart input-output connectivity maps of whole-brain circuits, offering invaluable insights into brain function and organization.

### 3.4 Applications of knowledge graphs in brain region connectivity

The application of the knowledge graph involves validating and discovering brain region connectivity knowledge, as well as retrieving literature pertaining to brain region connectivity relations. These connectivity relations denote the inputs and outputs of different neurons, and acquiring a comprehensive understanding of whole-brain connectivity relations is imperative for elucidating the inputs and outputs of the entire brain.

Previous studies (Bota *et al.* 2015, Swanson *et al.* 2017) have explored brain connectivity maps of limited brain regions, often based on experimental findings and report summaries. In contrast, the whole-brain connectivity knowledge graph synthesizes the input-output circuits of all brain regions documented in the literature. This facilitates researchers in efficiently verifying and analyzing the input-output circuits of any brain region.

**Figure 5.**
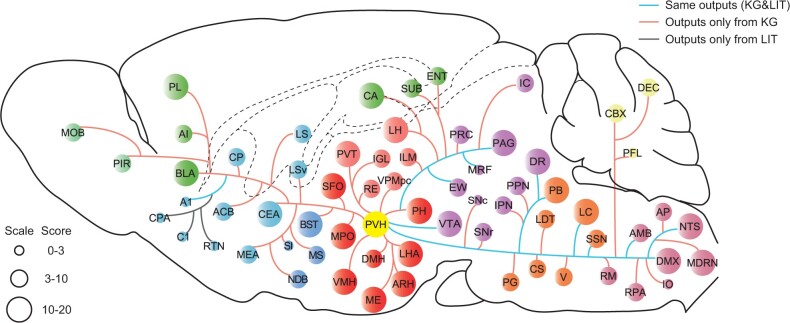
Connection diagram of PVH output circuit generated by the brain region connectivity knowledge graph. The linkages in the figure represent the existence of literature studying the projection of PVH brain regions to the corresponding brain regions. The different colors represent the functional groups to which the different brain regions belong, and the different circle sizes represent the heat of research on PVH output to that brain region.

Taking the paraventricular hypothalamic nucleus (PVH) as an example, Fig. 5 shows the output circuits originating from the PVH. The size of the circle represents the research heat of the PVH output to this brain region, as calculated by the TriModel knowledge graph embedding approach (Mohamed *et al.* 2021). This method achieves superior results in evaluating connectivity relation triplets, with further details provided in the Supplementary Materials. The PVH output scores are outlined in Supplementary Table S6. The line represents the PVH output obtained from different sources of information.

Brain regions with higher research heat of the PVH output include hypothalamus (HY): median eminence (ME), arcuate hypothalamic nucleus (ARH), lateral hypothalamic area (LHA), etc.; midbrain (MB): ventral tegmental area (VTA), dorsal nucleus raphe (DR), periaqueductal gray (PAG), etc.; medulla (MY): nucleus of the solitary tract (NTS), dorsal motor nucleus of the vagus nerve (DMX), medullary reticular nucleus (MDRN), etc.; thalamus (TH): lateral habenula (LH), paraventricular nucleus of the thalamus (PVT), ventral posteromedial nucleus of the thalamus, parvicellular part (VPMpc), etc.; striatum (STR): central amygdalar nucleus (CEA), nucleus accumbens (ACB), caudate putamen (CP), etc.; pons (P): locus ceruleus (LC), parabrachial nucleus (PB), laterodorsal tegmental nucleus (LDT), etc., and other brain regions bed nucleus of the stria terminalis (BST), basolateral amygdalar nucleus (BLA), prelimbic area (PL), etc. The PVH outputs are generally consistent with those of Geerling *et al.* (2010). The brain regions that include MB: VTA, DR, Edinger-Westphal nucleus (EW), periaqueductal gray ventrolateral division (PAGvl), and midbrain reticular formation (MRF); P: LC, PB; MY: NTS, DMX, superior salivatory nucleus (SSN), Compact and external formations of the nucleus ambiguus (NAc), and A1 have the same outputs. However, retrotrapezoid nucleus (RTN) and caudal pressor area (CPA) are not included in the PVH connectivity knowledge graph because their names are not in the existing nomenclature. In addition, caudal C1 catecholamine neuron (C1) is not recognized as a brain region in our method due to the variety of meanings of the name. All abbreviations of the brain regions mentioned are listed in Supplementary Table S7. These results underscore the efficacy of the whole-brain connectivity knowledge graph to efficiently and precisely map the input-output circuits of any brain region, thereby facilitating the validation and analysis of experimental data.

The brain region knowledge graph complements experimental knowledge significantly. In the AMBCA, 16 954 pairs of brain regions with connectivity relations and 28 415 brain regions lacking such relations are identified in the ipsilateral connection. The knowledge graph can compensate for missing connectivity information resulting from varying experimental conditions and species strains. Notably, the AMBCA indicates no connectivity relations between VTA to LH and PL, PL to ACB, DR to ACB, and CEA to BST. However, the knowledge graph reveals contrary evidence, showcasing projections from VTA glutamatergic neurons to LH, as well as from PL glutamatergic neurons to ACB (Table 5).

**Table 5.** Examples of literature on brain region connectivity knowledge graph.

Order	Source	Target	Sentence
1	VTA	LH	Role of glutamatergic projections from <s> ventral tegmental area </s> to <o> lateral habenula </o> in aversive conditioning.
2	PL	ACB	The <s> nucleus accumbens </s>, which is implicated in this process, receives glutamatergic inputs from the <o> prelimbic cortex </o> and midline regions of the thalamus.
3	DR	ACB	Substantial numbers of TH-immunoreactive cells in the <s> DR </s> were found to project to the <o> nucleus accumbens </o>.
4	VTA	PL	This was associated with a heightened shock-induced prelimbic cortex Fos response and activation of cholera toxin b retro-labeled <s> VTA </s> neurons that project to the <o> prelimbic cortex </o>.
5	CEA	BST	Rat <s> central amygdaloid nucleus </s> projections to the <o> bed nucleus of the stria terminalis </o>.

**Figure 6.**
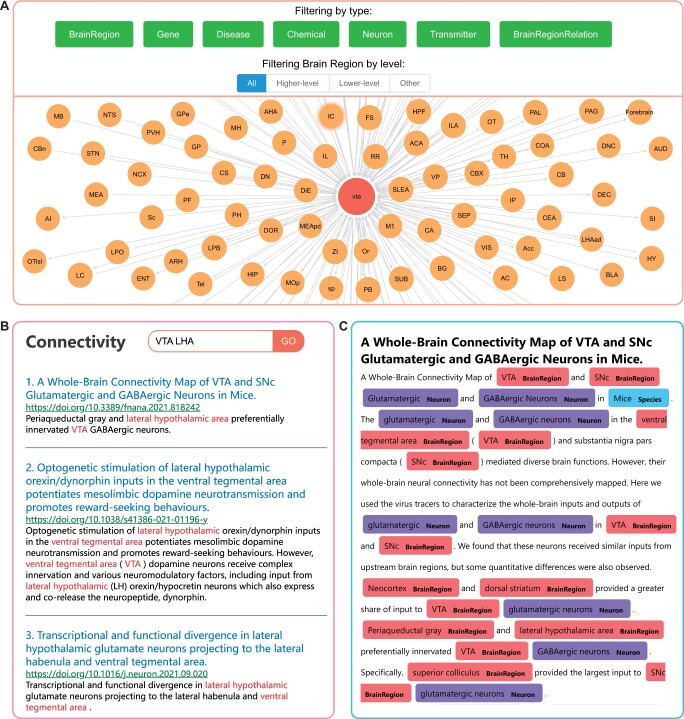
Brain region connectivity knowledge graph information. (A) shows the result for VTA in the text mining search tool. (B) and (C) show the abstracts and annotation information with connectivity relations obtained by brain region knowledge graph retrieval.

A text mining system has been developed based on the brain region connectivity knowledge graph, facilitating rapid search for brain region directional relations, document content, and highlighting. Functioning as a semantic search tool, it enables the retrieval of brain region directional relations. Initially, input words are matched against all abbreviations and synonyms within the previously established dictionary. By aggregating all matching names in the knowledge graph, comprehensive information regarding all input-output circuits of a single brain region is obtained, as shown in Fig. 6A. In addition, the system provides pertinent details such as brain region-specific literature titles, authors, journals, directional relations, and more. Within a single literature piece, different types of entities are differentiated using various colors, as shown in Fig. 6B and C. These entities are extracted from PubTator Central by providing the PMID for each article.

## 4 Conclusion

In this study, we undertake the extraction of brain regions, their connectivity, and directional relations from a large amount of literature, culminating in the construction of a literature-based knowledge graph depicting brain region connectivity. The knowledge graph serves as a valuable resource, facilitating researchers in swiftly searching and querying input-output circuits of various brain regions within the literature, thereby providing reliable verification and analysis of experimental data.

Our method builds upon and extends the work of French *et al.* (2009) and Richardet *et al.* (2015). Initially, we evaluate the effectiveness of several existing pre-trained models against the BioSEPBERT model in the tasks of NER and RE. The BioSEPBERT model significantly enhances the recognition of long words in brain regions by incorporating the position pointers and improves connectivity relation extraction through the introduction of special identifiers with self-attention. We achieve optimal F1 scores for both tasks with the BioSEPBERT model. Subsequently, we create a directional relation corpus based on the connectivity corpus through manual labeling, wherein the BioSEPBERT relation extraction model attains optimal results. Finally, we apply the BioSEPBERT model to extract knowledge from 1.3 million abstracts and 193 100 full-text articles, constructing a knowledge graph by obtaining brain regions, connectivity, and directional relations from the literature.

Despite our achievements, limitations remain. Firstly, the brain region NER is mainly restricted by the quantity and quality of the corpus. A more extensive and accurate corpus of brain regions must be created to enhance the model's performance. Secondly, automated methods currently lack the capability to extract connection strength hierarchy. A corpus of connection relations based on the strength hierarchy needs to be created. Lastly, gaining a comprehensive understanding of neuroscience literature necessitates additional knowledge of neurons and transmitters, for which a domain-specific dictionary can be used to build the corresponding corpus.

In the future, we aim to develop techniques for recognizing neuron and transmitter names in literature, thereby facilitating a deeper understanding of neuron types and their distribution across different brain regions.

## Supplementary Material

btae648_Supplementary_Data
