# Supplementary material for: Knowledge mining of brain connectivity in massive literature based on transfer learning
Source: Bioinformatics. 2024 Dec 5;40(12):btae648. doi: 10.1093/bioinformatics/btae648 (PMC11631446; doi:10.1093/bioinformatics/btae648)
Supplement: btae648_Supplementary_Data [file btae648_supplementary_data.pdf]

Supplementary material

Parameter Settings

All the models train on one NVIDIA Quadro RTX 5000 GPU. The environment configured for pre-trained models is Python 3.8.19 and Pytorch 1.8.1. The initial learning rate is 5×10^{-5}. The batch size is 16. The maximum length of the token is 512. The dropout size of the attention layer and hidden layer is 0.1. The optimization algorithm is Adam optimizer. The epoch is 3 for all cross-validation under different tasks. The environment configured for recurrent neural network models is Python 3.6.5 and Pytorch 0.2.0. The learning rate is 0.01 with a decay rate of 0.05. The dimensions of word and character embedding vectors are set to 200 and 30, respectively.

Hyperparameters Tuning

To identify the optimal hyperparameters, we evaluate various configurations of the baseline BioBERT model on the WhiteText dataset. We start with the default settings of the BioBERT model and fine-tune key parameters: batch size, learning rate, and epoch—factors known to significantly impact model performance. During the comparison of batch size and learning rate, the epoch is fixed at 3. As shown in Supplementary Table 1, the best performance is achieved with a batch size of 16 and a learning rate of 5×10^{-5}, resulting in an F1 score of 81.84%. Subsequently, we fix the batch size and learning rate and evaluate the model performance across epochs 1 through 5. The corresponding F1 scores are 77.96%, 80.45%, 81.59%, 79.33%, and 81.56%, respectively, with the highest performance observed at epoch 3, yielding an F1 score of 81.59%. These results indicate that the optimal hyperparameters for the BioBERT model on the WhiteText dataset are a batch size of 16, a learning rate of 5×10^{-5}, and an epoch of 3. These hyperparameters are consistently applied across all subsequent experiments to maintain comparability across models.

Supplementary Table 1 The F1 scores of the BioBERT model with different hyperparameters

Hyperparameter		Batch Size		
		16	32	64
Learning Rate	1×10^{-5}	81.03%	80.27%	80.21%
	3×10^{-5}	81.21%	81.49%	80.95%
	5×10^{-5}	**81.84**%	80.87%	80.27%

Performance of Various Models in Brain Region Named Entity Recognition (NER) and Relation Extraction (RE) Tasks

In the present study, we investigate the performance of different models in brain region NER and RE tasks within the WhiteText corpus. For the NER task, various models are employed, and the evaluation is conducted using 8-fold cross-validation in two ways: exact matching and lenient matching. The detailed results of these evaluations can be found in Supplementary Table 2. Moreover, we extend our evaluation to the WhiteText connectivity corpus, which is categorized into document-level and sentence-level corpora. The evaluation results of the models on both corpora

are delineated in Supplementary Table 3.

Supplementary Table 2 Comparative analysis of brain region NER models' performance against the WhiteText corpus. The data is represented as mean ± std

Model	Exact comparison			Lenient comparison		
	P(%)	R(%)	F1(%)	P(%)	R(%)	F1(%)
SOTA	82.10	81.50	81.80	91.60	85.70	88.60
MTM-CW	80.52±2.10	81.76±1.48	81.12±1.04	-	-	-
BioBERT	79.75±3.65	83.97±1.88	81.76±2.27	88.34±4.00	93.02±1.89	90.57±2.35
SciBERT	80.14±3.59	84.10±2.08	82.03±2.37	88.16±3.51	92.54±1.92	90.25±2.00
ClinicalBERT	78.78±3.67	83.21±2.04	80.89±2.36	87.46±4.21	92.36±1.37	89.79±2.36
PubMedBERT	80.92±3.49	84.81±2.05	82.79±2.45	88.78±2.90	93.08±2.03	90.85±1.80
BioSEPBERT	81.54±3.35	84.38±1.89	82.90±2.17	89.08±3.20	93.29±1.46	91.10±1.76
BioSEPBERT-DEN	**84.34**±2.57	**85.63**±1.42	**84.97**±1.83	**92.17**±2.26	**94.59**±0.78	**93.35**±1.20

Supplementary Table 3 Comparison of performance of brain region RE models against the WhiteText corpus. The data is represented as mean ± std

Model	WhiteText-Doc			WhiteText-Sen		
	P(%)	R(%)	F1(%)	P(%)	R(%)	F1(%)
SOTA	73.65	75.89	75.00	73.65	75.89	75.00
Att-BLSTM	67.69±4.04	63.74±5.61	64.44±4.65	74.96±1.88	70.53±4.14	72.08±2.78
BioBERT	77.47±3.58	72.69±9.09	74.73±5.32	**85.64**±2.04	84.10±2.45	84.84±1.84
SciBERT	75.26±3.47	76.21±6.43	75.63±4.19	83.85±2.26	84.69±1.98	84.25±1.61
ClinicalBERT	71.12±3.53	71.85±8.51	71.23±4.78	82.57±2.44	83.28±2.87	82.89±1.92
PubMedBERT	75.39±3.39	79.86±6.35	77.38±3.07	83.22±1.86	88.38±2.11	85.70±1.42
BioSEPBERT	**76.71**±3.25	**81.07**±4.09	**78.70**±1.58	84.90±2.87	**88.47**±2.25	**86.61**±1.63

Confusion Matrix in Directional Relation Extraction Task

To highlight the improvements achieved by our model in the directional relation extraction task, we compared the confusion matrices of the PubMedBERT and BioSEPBERT models, as shown in Supplementary Table 4. The optimal results from each model on the same dataset are selected for comparison, with 10 evaluations conducted due to the 10-fold cross-validation method used in this study. In Supplementary Table 4, the number of correct predictions made by PubMedBERT in each

category is 2866, 147, 205, and 86, corresponding to 98%, 84%, 86%, and 87% after normalization. In contrast, BioSEPBERT correctly predicted 2873, 142, 219, and 87 instances, with normalized values of 98%, 80%, 92%, and 88%, respectively. Although our method does not result in significant improvements in macro-F1 values, BioSEPBERT achieves a 7.0% and 1.1% improvement in output and input connections, respectively, demonstrating its superior ability to extract output and input connections from the literature.

Supplementary Table 4 Confusion matrix of the PubMedBERT and BioSEPBERT models in the directional relation extraction task. The four labels represent unconnected relation, undirected connection, output connection and input connection

True Label	PubMedBERT				BioSEPBERT			
	Neg	Connect	Output	Input	Neg	Connect	Output	Input
Neg	2866	29	24	8	2873	24	23	7
Connect	29	147	0	0	33	142	1	0
Output	28	0	205	5	17	0	219	2
Input	11	0	2	86	8	1	3	87

Evaluation of the Ontology Mapping Process

We evaluate the matching rates of two methods for linking entities to ontology on the WhiteText corpus (2,817 abstracts, 20,283 brain region entities). The results are presented in Supplementary Table 5. Using the exact matching method, two brain region ontologies—Allen, comprising 927 unique brain regions, and a merged ontology with 2,354 brain regions for mice and rats (integrating Allen, NeuroNames, and BAMS)—achieved match rates of 34.83% and 40.94%, respectively. In comparison, the Unified Medical Language System (UMLS), utilizing the ScispaCy tool, reached a higher match rate of 64.84%. In the exact matching process, brain regions are directly matched with terms in the ontology, so the matching rate corresponds to the accuracy rate. However, the highest accuracy rate for brain region ontologies is only 40.94%, limiting the effective utilization of identified information. Although UMLS shows a higher accuracy rate of 64.84%, it includes genes, diseases, and other entities, so the actual accuracy for brain region entities is lower than this figure suggests.

To improve matching accuracy, we develop a fuzzy matching method based on single-word matching and weighted sorting according to word-matching scores. This approach achieves an 84.02% matching rate on the WhiteText corpus, significantly enhancing the linkage of brain region entities to the ontology. Additionally, to verify the accuracy of this method, we randomly select 100 abstracts from the WhiteText corpus and manually compare 298 brain region entities against the ontology. We find that 261 entities are accurately matched, resulting in an accuracy rate of 87.58%. In statistics, the overall accuracy of a dataset can be estimated from the accuracy of a sample.

Supplementary Table 5 Evaluation of different ontology mapping process methods

Method	Ontology	Correct Brain Region Entity Number	Total Brain Region Entity Number	Match Rate
Exact	Allen	7,065	20,283	34.83%
	Merged Ontology	8,304		40.94%
	UMLS	13,151		64.84%
Fuzzy	Merged Ontology	17,042	20,283	84.02%

Additional Knowledge Graph Information

In our study, we employ the knowledge graph embedding method named TriModel to calculate the scores of PVH outputs to other brain regions. The TriModel facts as subject, predicate, and object (SPO) triples, where the subjects and objects are brain region entities, and the predicates represent brain region connectivity relations. The model generates negative samples by randomly corrupting the subjects and objects of the input true triplets. It then retrieves the corresponding embeddings for both the true and corrupted triplets. These embeddings are processed using model-specific scoring functions to generate scores for all the triplets. The objective is to maximize the scores of the true triplets while minimizing the scores of the corrupted ones. In our experiments, the data used in the TriModel method are extracted from 1.3 million abstracts and 193,100 full-text articles for input-output connectivity relations. The scores generated by the model reflect the heat of research focus on brain region connectivity relations, as documented in Supplementary Table 6. The abbreviations and full names of brain regions are cross-referenced with the Allen Mouse Brain Connectivity Atlas, the Brain Architecture Management System, and the Swanson nomenclature. Furthermore, all brain region abbreviations mentioned in the main text are listed in Supplementary Table 7.

Supplementary Table 6 The knowledge graph embedding score of the PVH output to the brain regions

Nomenclature	Areas	Regions		Knowledge graph embedding score
Allen	Cerebellum	DEC	Declive (VI)	9.22
		CBX	Cerebellar cortex	3.86
		CENT	Central lobule	3.09
		PFL	Paraflocculus	2.57
		FL	Flocculus	2.19
	Cortex	BLA	Basolateral amygdalar nucleus	11.79
		CA2	Field CA2	11.01
		PL	Prelimbic area	10.69
		ENT	Entorhinal area	6.78
		MOB	Main olfactory bulb	6.73
		SUB	Subiculum	5.23
		PIR	Piriform area	5.00
		AI	Agranular insular area	4.08
		CA3	Field CA3	2.97
		CA1	Field CA1	2.71
		CA	Ammon's horn	2.67
		DG	Dentate gyrus	2.38
		PRE	Presubiculum	2.05
		POST	Postsubiculum	2.00
	Hypothalamus	ME	Median eminence	19.96
		ARH	Arcuate hypothalamic nucleus	16.26
		PVH	Paraventricular hypothalamic nucleus	13.39
		LHA	Lateral hypothalamic area	13.25
		MEPO	Median preoptic nucleus	12.88
		SFO	Subfornical organ	12.24
		PH	Posterior hypothalamic nucleus	10.60
		VMH	Ventromedial hypothalamic nucleus	10.57
		MPO	Medial preoptic area	10.06
		DMH	Dorsomedial nucleus of the hypothalamus	9.83
		SO	Supraoptic nucleus	9.77
		ZI	Zona incerta	7.28
		SCH	Suprachiasmatic nucleus	6.28
		AVP	Anteroventral preoptic	5.09

			nucleus	
		OV	Vascular organ of the lamina terminalis	4.35
		PVHp	Paraventricular hypothalamic nucleus, parvicellular division	4.32
		RCH	Retrochiasmatic area	4.20
		STN	Subthalamic nucleus	3.86
		MEZ	Hypothalamic medial zone	3.80
		LPO	Lateral preoptic area	3.76
		PMv	Ventral premammillary nucleus	2.74
		SUM	Supramammillary nucleus	2.63
		TM	Tuberomammillary nucleus	2.43
		AVPV	Anteroventral periventricular nucleus	2.43
		VLPO	Ventrolateral preoptic nucleus	2.27
		ASO	Accessory supraoptic group	2.21
	Midbrain	VTA	Ventral tegmental area	18.09
		DR	Dorsal nucleus raphe	16.00
		PAG	Periaqueductal gray	12.39
		EW	Edinger-Westphal nucleus	7.35
		SNr	Substantia nigra, reticular part	5.00
		IPN	Interpeduncular nucleus	4.95
		PPN	Pedunculopontine nucleus	4.55
		IC	Inferior colliculus	4.53
		PRC	Precommissural nucleus	3.10
		SNc	Substantia nigra, compact part	2.60
		MEV	Midbrain trigeminal nucleus	2.07
	Medulla	NTS	Nucleus of the solitary tract	21.59
		DMX	Dorsal motor nucleus of the vagus nerve	13.28
		MDRN	Medullary reticular nucleus	12.45
		RPA	Nucleus raphe pallidus	8.12
		RM	Nucleus raphe magnus	5.85
		AMB	Nucleus ambiguus	5.40
		AP	Area postrema	4.37
		VII	Facial motor nucleus	2.59
		VI	Abducens nucleus	2.47
		IO	Inferior olivary complex	2.33

		CN	Cochlear nuclei	2.22
	Pons	LC	Locus ceruleus	18.55
		PB	Parabrachial nucleus	15.07
		CS	Superior central nucleus raphe	7.34
		LDT	Laterodorsal tegmental nucleus	6.58
		RPO	Nucleus raphe pontis	5.17
		B	Barrington's nucleus	5.08
		PG	Pontine gray	4.66
		V	Motor nucleus of trigeminal	4.00
	Pallidum	BST	Bed nuclei of the stria terminalis	17.03
		MS	Medial septal nucleus	6.05
		NDB	Diagonal band nucleus	3.94
		SI	Substantia innominate	2.63
		MA	Magnocellular nucleus	2.51
	Striatum	CEA	Central amygdalar nucleus	14.33
		ACB	Nucleus accumbens	9.47
		LSv	Lateral septal nucleus, ventral part	7.45
		CEAm	Central amygdalar nucleus, medial part	6.42
		MEA	Medial amygdalar nucleus	5.82
		LS	Lateral septal nucleus	5.72
		FS	Fundus of striatum	2.88
		STRv	Striatum ventral region	2.38
		CP	Caudoputamen	2.36
	Thalamus	LH	Lateral habenula	14.18
		PVT	Paraventricular nucleus of the thalamus	13.91
		VPMpc	Ventral posteromedial nucleus of the thalamus, parvicellular part	8.25
		RE	Nucleus of reunions	5.16
		IGL	Intergeniculate leaflet of the lateral geniculate complex	5.07
		ILM	Intralaminar nuclei of the dorsal thalamus	4.70
		Eth	Ethmoid nucleus of the thalamus	4.25
		VPL	Ventral posterolateral nucleus of the thalamus	3.78

		CL	Central lateral nucleus of the thalamus	2.95
		LGd	Dorsal part of the lateral geniculate complex	2.70
		VPM	Ventral posteromedial nucleus of the thalamus	2.66
		PIN	Pineal body	2.37
		PCN	Paracentral nucleus	2.34
		MH	Medial habenula	2.32
		RT	Reticular nucleus of the thalamus	2.14
BAMS	-	SSN	Superior salivatory nucleus	4.27
		RET	Reticular formation	2.42
		A1	A1	2.36

Supplementary Table 7 Abbreviation table for brain regions in the main text

Nomenclature	Abbreviation	Brain region names
Allen	ACB	Nucleus accumbens
	AI	Agranular insular area
	AMB	Nucleus ambiguus
	AP	Area postrema
	ARH	Arcuate hypothalamic nucleus
	BLA	Basolateral amygdalar nucleus
	BST	Bed nuclei of the stria terminalis
	CA	Ammon's horn
	CBX	Cerebellar cortex
	CEA	Central amygdalar nucleus
	CP	Caudoputamen
	CS	Superior central nucleus raphe
	DEC	Declive (VI)
	DMH	Dorsomedial nucleus of the hypothalamus
	DMX	Dorsal motor nucleus of the vagus nerve
	DR	Dorsal nucleus raphe
	ENT	Entorhinal area
	EW	Edinger-Westphal nucleus
	HY	Hypothalamus
	IC	Inferior colliculus
	IGL	Intergeniculate leaflet of the lateral geniculate complex
	ILM	Intralaminar nuclei of the dorsal thalamus
	IO	Inferior olivary complex
	IPN	Interpeduncular nucleus
	LC	Locus ceruleus
	LDT	Laterodorsal tegmental nucleus
	LH	Lateral habenula
	LHA	Lateral hypothalamic area
	LS	Lateral septal nucleus
	LSv	Lateral septal nucleus, ventral part
	MB	Midbrain
	MDRN	Medullary reticular nucleus
	ME	Median eminence
	MEA	Medial amygdalar nucleus
	MOB	Main olfactory bulb
	MPO	Medial preoptic area
	MS	Medial septal nucleus
	MY	Medulla
	NDB	Diagonal band nucleus

	NTS	Nucleus of the solitary tract
	P	Pons
	PAG	Periaqueductal gray
	PAGvl	Periaqueductal gray ventrolateral division
	PB	Parabrachial nucleus
	PFL	Paraflocculus
	PG	Pontine gray
	PH	Posterior hypothalamic nucleus
	PIR	Piriform area
	PL	Prelimbic area
	PPN	Pedunculopontine nucleus
	PRC	Precommissural nucleus
	PVH	Paraventricular hypothalamic nucleus
	PVT	Paraventricular nucleus of the thalamus
	RE	Nucleus of reunions
	RM	Nucleus raphe magnus
	RPA	Nucleus raphe pallidus
	SFO	Subfornical organ
	SI	Substantia innominate
	SNc	Substantia nigra, compact part
	SNr	Substantia nigra, reticular part
	STR	Striatum
	SUB	Subiculum
	TH	Thalamus
	V	Motor nucleus of trigeminal
	VMH	Ventromedial hypothalamic nucleus
	VPMpc	Ventral posteromedial nucleus of the thalamus, parvicellular part
	VTA	Ventral tegmental area
BAMS	A1	A1
	C1	Caudal C1 catecholamine neurons
	MRF	Midbrain reticular formation
	SSN	Superior salivatory nucleus
Swanson	CPA	Caudal pressor area
	NAc	Compact and external formations of the nucleus ambiguus
	RTN	Retrotrapezoid nucleus
